# Difference among frailty assessment tools in predicating postoperative prognosis of elderly patients with mild traumatic brain injury

**DOI:** 10.1016/j.clinsp.2024.100554

**Published:** 2025-01-25

**Authors:** Chunhua Ni, Chen Gu, Hua Liu, Feng Cheng, Chao Cheng, Xiaohua Xia

**Affiliations:** a Intensive Care Unit, Kunshan Hospital Affiliated to Jiangsu University, Qianjin East Road, China; b Neurosurgery Department, Kunshan Hospital Affiliated to Jiangsu University, Qianjin East Road, China; c Emergency Department, Kunshan Hospital Affiliated to Jiangsu University, Qianjin East Road, China

**Keywords:** Elderly population, Clinical frailty scale, Frailty index, Glasgow outcome scale, Mild Traumatic Brain Injury

## Abstract

**Objectives:**

Mild Traumatic Brain Injury (mTBI) is quite prevalent in the elderly population, and the authors performed a retrospective analysis regarding the predictive value of frailty assessing tools regarding the prognosis of elderly mTBI patients.

**Methods:**

All the patients underwent assessment of frailty upon admission using five tools including Frailty Phenotype (FP), FRAIL Scale (FS), Edmonton Frailty Scale (EFS), Groningen Frailty Indicator (GFI), and Clinical Frailty Scale (CFS). The predicting potential of tools was analyzed against the prognosis defined by the extended Glasgow Outcome Scale (GOSE).

**Results:**

The incidence of frailty in elderly patients varies widely among the tools. Multivariate logistic regression analysis showed that only frail conditions defined by FP (p-value = 0.014) and FS (p-value = 0.004) could be employed for predicting unfavorable prognosis defined by GOSE, while frailty defined by CFS (p-value = 0.683), EFS (p-value = 0.301) and GFI (p-value = 0.925) could not. The ROC further showed that FP (AUC = 73.2 %) and FS (AUC = 76.2 %) had moderate power in predicting unfavorable conditions, while CFS (AUC = 46.1 %), EFS (AUC = 55.6 %), and GFI (AUC = 51.5 %) only had low or even no power.

**Conclusions:**

FP and FS could be used to predict the unfavorable prognosis associated with mTBI in the elderly population.

## Highlights

• Incidence of frailty in elderly patients varies widely among different tools.
• FP and FS could be employed as tools for predicting frail conditions.
• Different tools had low consistency in predicting frail conditions.

## Introduction

Traumatic Brain Injury (TBI) generally refers to damages such as intracranial hemorrhage or skull fracture caused by indirect or direct physical or mechanical external force impacts, and will finally lead to brain dysfunction in patients.^1^ TBI is now being recognized as a global issue that affects around 100–300 hospitalized patients per 100,000 people annually.^2^ Since acute management and healthcare have been recommended in recent decades, the death rate for TBI patients has considerably decreased.^3^^,^^4^ Nonetheless, a variety of persistent disorders, such as deficits in mental, physical, behavioral, emotional, and social functioning, can still arise subsequently.^5^^,^^6^ Of different TBI types, mild Traumatic Brain Injury (mTBI) accounts for 80 %–90 % of all the cases, and due to the rapidly aging population, the number of older people suffering from mTBI is continuously rising.^7^ The collaborative European Neurotrauma Effectiveness Research (CENTER-TBI), a multicenter European-based cohort study of over 4500 patients, indicated that 28 % of the population was older than 65 years, and the major factor of TBI in this population is low velocity falls.^8^^,^^9^ Despite the rising prevalence of mTBI in elderly patients, very few studies have focused exclusively on the conditions in this patient population.

In recent years, emerging attention has been focused on pre-injury functional status, as a possible further prognostic factor. A number of epidemiological studies have determined that male sex, youth, poor socioeconomic status, pre-existing mental health disorders, and history of head trauma are significant risk factors for TBI.^10^ Even though the majority of these pre-injury risk variables have undergone extensive evaluation, it is still imperative to determine the risk factors that impact the prognosis of TBI patients. Frailty is a multifaceted syndrome marked by a reduced physiological reserve and a heightened susceptibility to unfavorable health outcomes in the context of medicine.^11^ The term has been employed to describe the state in which a person is more susceptible to unfavorable events such as falls, disability, hospitalization, and/or mortality due to age-related decreases in several physiological systems.^11^, ^12^, ^13^, ^14^ Frailty is currently assessed in a variety of ways. The term “Frailty Index (FI)” can be either produced using a system based on a “deficit accumulation model”, which assesses comorbidities, medications, and laboratory abnormalities, or produced based on a multidimensional biopsychosocial model, which combines physical and psychosocial domains.^15^ However, due to the lack of a unified concept of frailty, researchers from various countries have developed a large number of induces based on different conceptual models. Among them, the Frailty Index (FI) and Frailty Phenotype (FP) are the two most widely used worldwide, and often used as a calibration standard to test the performance of other tools.^16^ Other frailty assessment tools include the FRAIL Scale (FS), the Clinical Frailty Scale (CFS), the Groningen Frailty Indicator (GFI), and the Edmonton Frailty Scale (EFS). These tools are important in evaluating dimensions, generalization, and are different in the frame of reading, the way of measurement, etc. However, which tool is more suitable for assessing frailty in elderly mTBI patients is not determined.

In the current study, the authors performed a retrospective analysis to evaluate the potential of five different FIs in predicting the prognosis of elderly patients with mTBI. By comparing the applicability of different frailty assessment tools, the current study attempted to provide valuable information for these patients in choosing the right tool and achieving the goal of reversing the frail state and improving the prognosis of surgery.

## Methods

### Patients

The retrospective analysis involved 301 participants collected from June 2020 to May 2023 in Kunshan Hospital Affiliated with Jiangsu University, and all the participants should be 55 years or older, having sustained mTBI symptoms such as loss of consciousness and/or posttraumatic amnesia, after a diagnosis of moderate-severe TBI (Glasgow Coma Scale [GCS ≤13]) and CT scans. Exclusion criteria were insufficient comprehension of language, a history of brain injury or psychiatric disorder, substance use disorders, inability to comply with follow-up, suspected neurodegenerative disease, etc. All the patients had signed a written informed consent form. All the analysis procedures were performed in accordance with the Declaration of Helsinki and following the STROBE Statement, and were performed in compliance with relevant laws and institutional guidelines approved by the ethics committee of Kunshan Hospital Affiliated with Jiangsu University for the related screening, inspection, and data collection (n° 2023–03–057-K01, 2023.11.9).

### Collection of clinicopathological information

A general information questionnaire was developed by the investigators based on the literature review. The questionnaire includes both sociodemographic data and disease-related information, including age, gender, comorbidities, premental health, loss of consciousness, intracranial injuries, and cause of injuries. CT scans of the brain were routinely taken in the first 24 h after the admission. The prognosis of the patients was evaluated after six months using the extended Glasgow Outcome Scale (GOSE) and was dichotomized into unfavorable (score 1–3) and favorable (score 4–8) outcomes following previously published works.^17^, ^18^, ^19^, ^20^, ^21^

### Frailty assessment tools

The frailty was assessed within 24 h after the admission, and for patients with communicating disorders, the assessment was performed by family members. In the current analysis, five assessing tools were used.

### Frailty phenotype (FP)

FP is a tool proposed by Fried in 2001,^12^ and is currently the most widely used frailty assessing toll in clinics. The tool is developed based on a frailty cycle model that evaluates older adults from the following five aspects: 1) Weight loss due to unknown reason: unexpected body weight loss in the last one-year (> 4.5 kg or > 5.0 %); 2) Fatigue: based on the assessment using the Center for Epidemiologic Studies Depression Scale (CES-D). Entry 1: the patient feels like it takes effort to do everything, Entry 2: I can't get up and do things. The occurrence of any entry three times or more in the last week was diagnosed as fatigue; 3) Low grip strength: measurement of grip strength twice with the dominant hand and the greater value is recorded. Low grip strength is assessed based on the corresponding age and gender using the Chinese Expert Consensus on Frailty Assessment and Intervention in Elderly Patients; 4) Slowing down of pace: measurement of the time taken to walk 4.57 m. Slowing down of pace is assessed according to the corresponding height and gender; 5) Low physical activity: for males < 383 kcal/week, women < 270 kcal/week is considered to be low based on the International Short Physical Activity Questionnaire was used (International Physical Activity Questionnaire Short Form, IPAQ-SF). The score is recorded as 1 if one of the above five aspects is met. The total score is 0∼5 points, with 0 points being no frailty, 1∼2 points being pre-frailty, and ≥ 3 points being frailty.

### FRAIL scale (FS)

This scale is a self-report scale proposed by the International Association on Nutrition and Aging in 2008^22^ and includes 5-items: 1) Fatigue: feeling tired most or all of the time in the past four weeks; 2) Increased resistance/endurance: difficulty in ascending and descending stairs independently; 3) Decreased free mobility: limited walking for 1 block (100 m) independently; 4) Disease status: five or more chronic diseases, 5) Weight loss: weight loss of ≥ 5 % in one year or less. One point is scored for meeting 1 criterion, and the total score is 0∼5 points. 0 points represent no frailty, l∼2 points represent pre-frailty, and ≥ 3 points are defined as frailty.

### Clinical frailty scale (CFS)

The scale is a 9-level rating scale developed by a Canadian research institute.^23^ After assessing the patient's general condition, the patient is graded according to the severity of the disease, energy, physical activity, and physical function, and is classified into 9 grades from very healthy to the terminal stage. Each band on the scale corresponds to a written description of frailty, supplemented by a visual chart to help classify frailty and represent the assessor's overall impression of the elderly patient. In this study, grades 1∼4 are classified as no frailty, grades 5∼7 represent frailty (grade 5 mild frailty, grade 6 moderate frailty, and grade 7 severe frailty), and grade 8∼9 is near death.

### Edmonton frailty scale (EFS)

The scale was developed by Rolfson in 2006,^24^ and is also known as the “Edmund Frailty Scale”. The scale includes a total of 9 dimensions of cognitive ability, health status, functional dependence, social support, drug use, nutrition, mood, incontinence, and functional performance, with a total of 11 items. There are 2 objective tests (the clock-drawing test and the timed stand-up walking test). The total score is 0∼17 points, 7∼8 is mild frailty, 9∼10 is moderate frailty, and 11∼17 is severe frailty, the higher the score, the higher the degree of frailty.

### Groningen frailty indicator (GFI)

The scale is a standardized questionnaire covering individual multifunctional fields. The scale assesses the individual's absence of four functional dimensions: physical dimension (mobility, physical health, vision, hearing, nutrition and number of medications), cognitive dimension (cognitive function), social dimension (emotional isolation), and psychological dimension (depression and anxiety). The questionnaire includes a total of 15 items that are scored by the dichotomous method: 1 point indicates that the item problem exists, and 0 points indicate non-existent. The total score is 0∼15 points, higher scores indicate more severe frailty in the elderly, and ≥ 4 points are defined as frailty.

### Statistical analysis

The general data of patients and the assessment results of the five frailty tools were described according to the type of variables. Normally distributed data were represented as the mean ± Standard Deviation (SD), while non-normally distributed data were represented as the median and interquartile range (P25, P75). Frequency and percentage were used for counting data. The Kappa test was used to compare the consistency between the five tools: Kappa values of 0∼0.20, 0.21∼0.40, 0.41∼0.60, 0.61∼0.80, and 0.81∼1, represented very low, low, medium, high, and very strong agreement, respectively. Pearson's Chi-Square test, continuously corrected Chi-Square test, and Fisher's exact test was used to compare the differences between the counting data, and the Mann-Whitney *U* test in the nonparametric test was used to compare the differences between the grade data. Logistic regression and Receiver Operating Curve (ROC) methods were employed to assess the predictive value of different tools regarding the unfavorable status of the patients. The difference regarding Area Under Curve (AUC) values of different FIs was compared using a bootstrap method. The statistical analyses and graph plotting were conducted either using GraphPad Prism version 8.0.0 for Windows (GraphPad Software, San Diego, California USA, www.graphpad.com) or using *R* language version 4.2.2 with a significant level of 0.05.

## Results

### Participant characteristics

A total of 301 patients with mTBI aged from 55 to 88 years at the time of injury were assessed (68.2 ± 6.5). Demographic characteristics, and clinical and radiological parameters of patients receiving different frailty assessments were presented in Table 1. The prognosis of the patients was assessed using GOSE, and based on the criteria, 83 patients were classified as unfavorable status and 218 patients were classified as favorable status.

**Table 1:** Clinicopathological information.

	Value
Age (year)	68.2 ± 6.5
Male (n [%])	158 (52.5 %)
Comorbidities (n [%])	164 (54.5 %)
Premental health (n [%])	43 (14.8 %)
Glasgow Coma Scale	
13	41
14	86
15	174
Intracranial injuries (CT) (n [%])	110 (36.5 %)
Cause of injury [n(%)]	
Fall	229
Traffic	33
Violence	13
Other	26
GOSE	
Unfavorable	83
Favorable	218

### Frailty assessing results of different tools

The frailty conditions in elderly mTBI patients evaluated by the five tools are shown in Table 2. The incidence of frailty in elderly patients varies widely, and the incidence of frailty assessed by GFI was the highest 33.3 %, followed by CFS (22.2 %), FP (19.8 %), FS (11.6 %), and EFS (10.1 %). The incidence of pre-frailty assessed by FP and FS was 55.1 % and 53.6 %, respectively. Most of the patients assessed as frailty by CFS and EFS were mildly frail (Table 2). Regarding the consistency analysis, FP and FS, FS and GFI, CFS and GFI had statistically significant moderate consistency, while each other two tools had low consistency or no-consistency (Table 3).

**Table 2:** Frailty condition of the patients assessed by different tools.

**Tools**	**Number (n)**	**Proportion (%)**
FP		
Frailty (≥ 3)	74	24.6
Prefrailty (1∼2)	176	58.5
No frailty (0)	51	16.9
FS		
Frailty (≥ 3)	79	26.3
Prefrailty (1∼2)	153	50.8
No frailty (0)	69	22.9
CFS		
Severe frailty (level 7)	3	1.0
Moderate frailty (level 6)	24	8.0
Mild frailty (level 5)	42	14.0
No frailty (≤ level 4)	232	77.0
EFS		
Severe frailty (≥ 11)	12	4.0
Moderate frailty (9∼10)	14	4.6
Mild frailty (7∼8)	19	6.3
No frailty (≤ 6)	256	85.1
GFI		
Frailty (≥ 4)	105	34.9
No frailty (≤ 3)	196	65.1

**Table 3:** Consistent analysis between different assessing tools (Kappa-value).

	FP	FS	CFS	EFS	GFI
FP	1				
FS	0.518*	1			
CFS	0.312*	0.051	1		
EFS	0.267*	0.064	0.079	1	
GFI	0.176*	0.441*	0.396*	0.039	1

### Comparison of assessing characteristics of the five frailty assessment tools

Furthermore, the current analysis used CFS, which has a more granular grading of frailty, as a reference, to compare the assessing characteristics of different tools. The results showed that the other four tools classified the patients with severe frailty based on CFS to be frail (100 % frailty rate), indicating that the evaluation results of the five assessment tools were consistent when the degree of frailty was severe (Table 4). For patients with moderate frailty assessed by CFS, both FP and GFI classified those patients as frailty (100 % detection rate), but FS and EFS identified over 90 % of patients as frail and less 10 % as non-frail, indicating that when the degree of frailty was moderate, the assessing results of the five assessment tools were somewhat different, but basically the same (Table 4). For patients with mild frailty assessed as CFS, there was a large difference (23.9 %∼69.0 %) among the other four tools to determine frailty, indicating that when the patients were mildly frail, the evaluation results of the five frailty assessment tools were quite different (Table 4).

**Table 4:** Assessing characteristics of the five frailty assessment tools.

		**CFS**			
		**Severe frailty** **(*n* = 3)**	**Moderate frailty** **(*n* = 24)**	**Mild frailty** **(*n* = 42)**	**No frailty** **(*n* = 232)**
**FP**	Frailty	3 (100.0 %)	24 (100.0 %)	22 (52.4 %)	10 (4.3 %)
	Prefrailty	0 (0.0 %)	0 (0.0 %)	16 (38.1 %)	144 (62.1 %)
	No frailty	0 (0.0 %)	0 (0.0 %)	4 (9.5 %)	78 (33.6 %)
**FS**	Frailty	3 (100.0 %)	22 (91.7 %)	11 (26.2 %)	5 (2.2 %)
	Prefrailty	0 (0.0 %)	2 (8.3 %)	29 (69.0 %)	126 (54.3 %)
	No frailty	0 (0.0 %)	0 (0.0 %)	2 (4.8 %)	101 (43.5 %)
**EFS**	Severe frailty	3 (100.0 %)	5 (20.8 %)	1 (2.4 %)	0 (0.0 %)
	Moderate frailty	0 (0.0 %)	8 (33.3 %)	2 (4.8 %)	4 (1.7 %)
	Mild frailty	0 (0.0 %)	10 (41.7 %)	7 (16.7 %)	15 (6.5 %)
	No frailty	0 (0.0 %)	1 (4.2 %)	32 (76.2 %)	213 (91.8 %)
**GFI**	Frailty	3 (100.0 %)	24 (100 %)	29 (69.0 %)	48 (21.1 %)
	No frailty	0 (0.0 %)	0 (0.0 %)	13 (31.0 %)	183 (78.9 %)

### Correlation of five frailty assessing tools with the prognosis of elderly mTBI patients

**Fig. 1:**
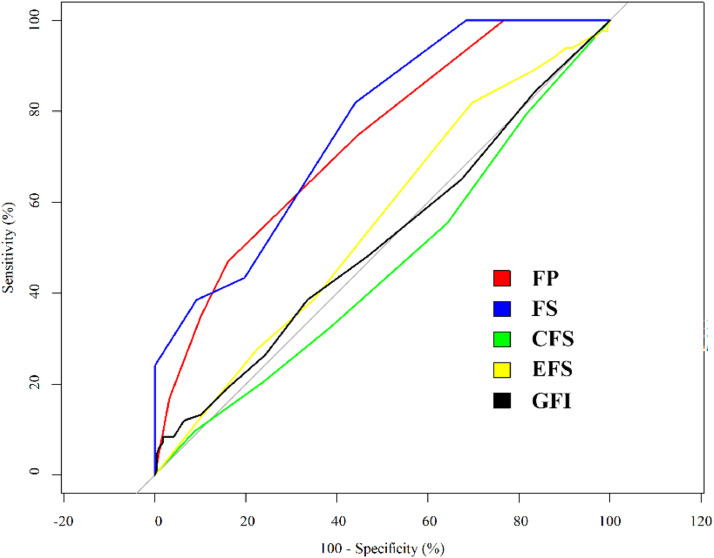
ROC analysis results of the predictive value of the five tools regarding the prognosis of elderly mTBI patients defined by GOSE.

The prognosis assessed by GOSE was used as the dependent variable, and the “frailty” defined by the five frailty assessing tools was used as a dichotomous self-variation. The results showed that when the variables with statistical significance in the univariate analysis were not corrected, the frailty defined by FP, FS or GFI was a risk factor for unfavorable prognosis, and the OR values of FP, FS, CFS, EFS and GFI were 3.80, 2.16, 4.99, 3.05, and 3.01, respectively (Table 5). Multivariate logistic regression analysis showed that frailty defined by FP and FS was still a risk factor for unfavorable prognosis, with OR values of 2.80 and 1.17, respectively, while frailty defined by CFS, EFS, and GFI were no longer correlated with unfavorable prognosis, and the difference was not statistically significant (*p* > 0.05) (Table 5). The results of logistic regression were further verified with ROC analysis. The results showed that FP (AUC = 73.2 % [95 % CI 67.0 %∼83.9 %]) and FS (AUC = 76.2 % [95 % CI 66.0 %∼81.9 %]) had moderate power in predicting the occurrence of unfavorable conditions defined by GOSE (Fig. 1), while CFS (AUC = 46.1 % [95 % CI 9.6 %∼91.3 %]), EFS (AUC = 55.6 % [95 % CI 30.3 %∼81.9 %]), and GFI [AUC = 51.5 % [95 % CI 8.4 %∼98.2 %]) only had low or even no predictive potential regarding the unfavorable conditions defined by GOSE (Fig. 1). The comparison of the AUC values of different FIs showed that no significant difference was detected between AUC values of FP and FS, which were both significantly larger than AUC values of CFS, EFS, and GFI (Table 6).

**Table 5:** Correlation between tools and prognosis defined by GOSE.

	**Univariate**			**Multivariate**		
	**OR value**	**95 % CI**	**p-value**	**OR value**	**95 % CI**	**p-value**
**FP**	3.80	3.68‒2.95	0.013	2.80	1.47‒1.96	0.014
**FS**	2.16	2.97‒3.38	0.015	1.17	0.97‒1.41	0.004
**CFS**	4.99	4.84‒5.16	0.901	2.97	2.82‒3.14	0.683
**EFS**	3.05	3.96‒4.14	0.079	2.05	2.96‒2.14	0.301
**GFI**	3.01	3.93‒4.10	0.008	1.00	0.91‒1.09	0.925

**Table 6:** Comparison of AUC values between different FIs in predicting the unfavorable condition defined by GOSE.

**FIs**	**AUC difference (%)**	**p-value**
FP∼FS	−2.95	0.460
FP∼CFS	27.11	<0.001
FP∼EFS	17.65	<0.001
FP∼GFI	21.74	<0.001
FS∼CFS	30.07	<0.001
FS∼EFS	20.06	<0.001
FS∼GFI	24.69	<0.001
CFS∼EFS	−9.47	0.071
CFS∼GFI	−5.37	0.301
EFS∼GFI	4.09	0.413

## Discussion

TBI in the elderly is mostly caused by falls and traffic accidents, and early diagnosis and treatment is the key to improve the prognosis. However, the assessment of frailty in elderly patients with mTBI has been complicated, both due to the difference in frailty assessing tools and incompliance in elderly people. To provide more valuable information for selecting optimal assessing tools in elderly patients with mTBI, the current study performed a retrospective analysis regarding 301 patients in the hospital, all of whom were diagnosed as mTBI and received assessments of frailty by different tools. Based on the analysis, it was found that the sensitivity varied greatly between different tools with GFI showing the highest sensitivity. Moreover, the consistency between different tools were overall low in predicting different frail states. However, based on the logistic regression and ROC analyses, only frailty defined by FP and FS were risk factors for unfavorable prognosis of elderly patients with mTBI and could be employed as potential predictive tools for the unfavorable conditions of the patients defined by GOSE.

Age is one of the predictors of poor prognosis in older patients with TBI. Recent studies have found that frailty is more reflective of the aging of the body and its tolerance to stressors such as trauma and surgery than age itself. Frailty is not a direct result of aging, instead it is often caused by age-related decline intersecting with chronic diseases.^25^ Frailty is a predictor of poor prognosis for several stressors, such as burns^26^ and general trauma,^27^ and has a persistent effect on the survival rate of elderly trauma patients at one year after injury, regardless of trauma severity or age.^28^ The study by Lee et al.^29^ suggested that frailty is an independent predictor of 30-day mortality in patients with mild to moderate TBI and a poor 6-month prognosis in patients with mTBI. Moreover, frailty is also associated with an increased risk of hospital-acquired infections after emergency surgery and a longer hospital stay in patients with TBI, and the impact of frailty is more pronounced in patients with mild to moderate diseases.^30^

According to the Chinese Expert Consensus on Frailty Assessment and Intervention in Elderly Patients, a good frailty assessment tool should not only be able to accurately identify frailty, but should also have the ability to predict response to treatment and negative clinical events (e.g., death) in elderly patients. Previous systematic reviews and related reviews have shown that, despite the influence of different assessment tools, frailty is generally an independent risk factor for adverse postoperative outcomes in elderly patients with mTBI, which can lead to increased morbidity, readmission rates, mortality, and decreased physical function. Similar conclusion was also drawn by the current study, which solidly affirmed the role of frailty in predicting the unfavorable conditions of patients included in the study. In addition, the current study also found that FP and FS, which focused on the evaluation of physical function, had a moderate predictive power of postoperative complications. On the contrary, for tools focusing on the multidimensional including physical, psychological, social, and cognitive functions, the poor predictive potential was shown, suggesting that the physical dimension of frailty is more associated with short-term postoperative outcomes (complications) than other dimensions. Moreover, as a single-dimensional assessment tool that also focuses on physical function, FS has items similar to FP but didn't show similar predictive power, suggesting that subjectively reported tools may be biased by overestimating/underestimating their physical condition in older patients and affect the results. The implications of these findings for clinical practice are significant: selecting FP and FS as primary assessment tools may facilitate early identification of high-risk elderly mTBI patients, enabling targeted interventions to mitigate risk and improve outcomes. However, it is crucial to recognize that this study's single-center, retrospective design and reliance on GOSE as the sole outcome metric may limit generalizability. Additionally, due to the heterogeneity of study subjects, this study only focuses on elderly mTBI patients, and it is not yet known whether this conclusion can be extrapolated to the entire TBI population. Future research should consider multi-center, prospective studies with a broader array of outcome measures to validate these findings and further elucidate the relative contributions of different frailty dimensions in mTBI prognosis.

Collectively, the study showed FP and FS emerge as robust tools for predicting adverse outcomes in elderly mTBI patients, reinforcing the value of frailty assessment in trauma care for older adults. Integrating these tools into clinical pathways may enhance risk stratification and outcome prediction, ultimately contributing to more individualized and effective management strategies in this vulnerable population. To promote the application of the conclusion of the current study, multi-center prospective studies using more direct endpoint measurements will be performed in the future.

## Authors’ contributions

CHN and CG performed conceptualization, data curation, formal analysis, and writing-original draft; HL and, FC, and CC performed conceptualization, data curation; XHX performed conceptualization and writing-review & editing.

## Funding

The 10.13039/501100002703current study is supported by Jiangsu University's 2023 Medical Education Collaborative Innovation Funding (n° JDYY2023055).

## Declaration of competing interest

The authors declare no conflicts of interest.

## Appendix Supplementary materials

Image, image 1
