# Supplementary figures and images for: Difference among frailty assessment tools in predicating postoperative prognosis of elderly patients with mild traumatic brain injury
Source: Clinics (Sao Paulo). 2025 Jan 25;80:100554. doi: 10.1016/j.clinsp.2024.100554 (PMC12013161; doi:10.1016/j.clinsp.2024.100554)

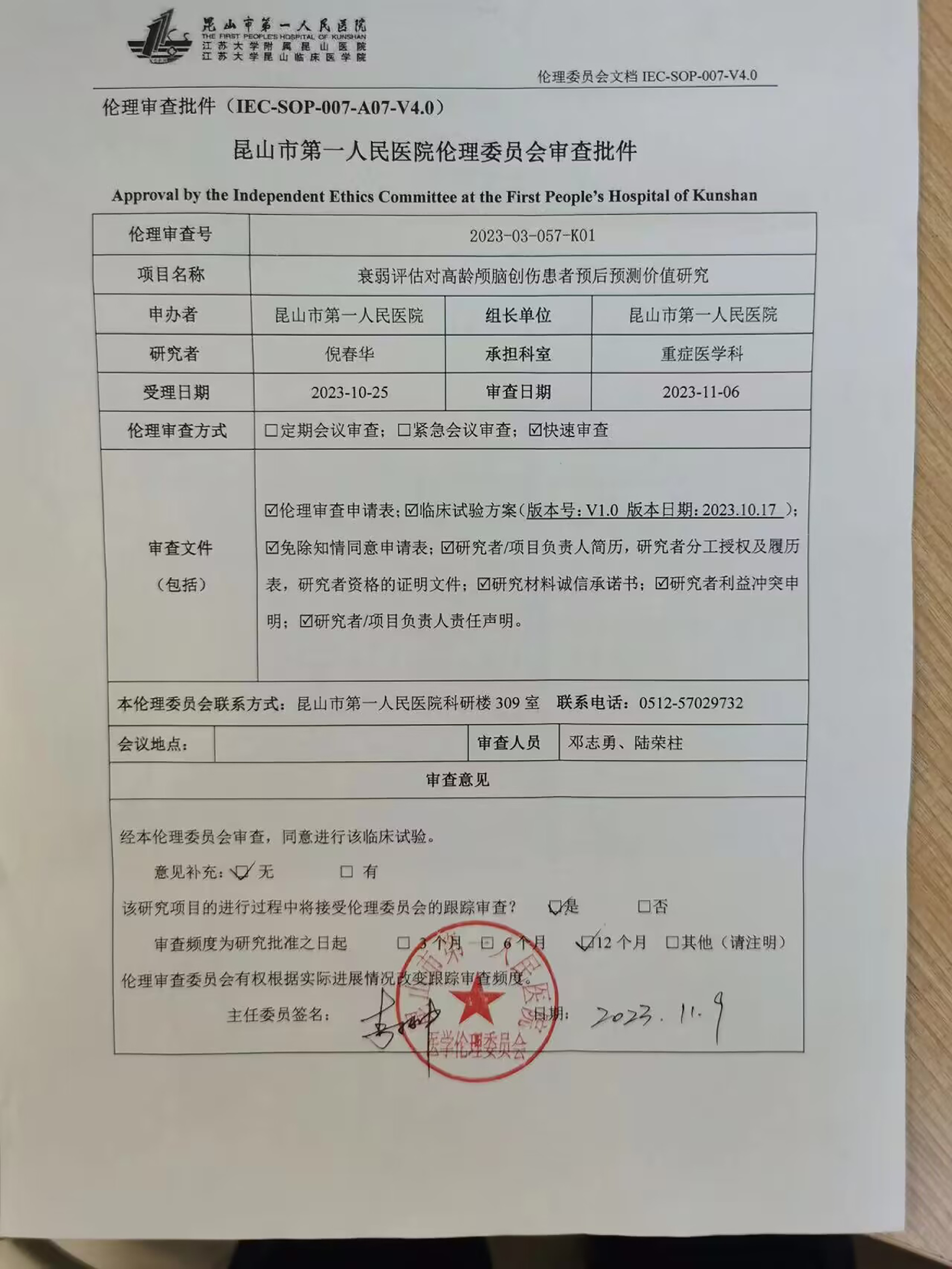

Supplement: Supplementary file 1 [file mmc1.jpg]
